# Ovarian Tumors

**Published:** 1861-12

**Authors:** Wm. H. Byford

**Affiliations:** Professor of Obstetrics and Diseases of Women and Children, in the Medical Department, Lind University, Chicago, Illinois


### CHICAGO MEDICAL EXAMINER

N. S. DAVIS, M. D., Editor.---FRANK W. REILLY, M. D., Ass't Editor.

VOL. II.] DECEMBER, 1861. [NO. 12.

### Original Contributions.

ARTICLE I.

### OVARIAN TUMORS.

By Wm. H. BYFORD, A. M., M. D.,

PROFESSOR OF OBSTETRICS AND DISEASES OF WOMEN AND CHILDREN, IN THE MEDICAL DEPARTMENT, LIND UNIVERSITY, CHICAGO, ILLINOIS.

### I.—Is Ovariotomy a Justifiable Operation?

This question is one which may be considered open for discussion, many eminent members of the profession, both in this country and in Europe, entirely rejecting it, while quite as many, perhaps, of equally good authority, advocate its adoption among the legitimate modes of relief in appropriate cases. The statistics of operations performed in the last few years, particularly by men who have endeavored to perfect it, and to make a just adaptation of it to cases, furnish its advocates with, I think, valuable proof of its beneficent effects.

Ovariotomy is comparatively a new operation; and, as yet, is not in that state of perfection that almost all other capital operations have attained; so that, in forming an opinion of its rate of mortality, as compared with other operations, we should make due allowance for short-comings in this respect. This remark, of course, is more strongly applicable to operations performed five, ten, fifteen, and even thirty years ago. The causes of failure then, were not so well understood as now, (nor now, as they will doubtless be in the future), nor were they avoided with the skill we may hope some time to employ. There are but few of the other capital operations which have not arrived at perfection, so far as their mechanical execution is concerned, and perhaps the prior and after treatment of the case for which they are performed. They consequently have the great advantage, thus arising, in any comparison of value or safety. The statistics of ovariotomy include cases treated by all sorts of surgeons, under all sorts of circumstances, during the whole time from its initiation in 1809, by Dr. McDowell, of Kentucky, up to the present time; thus presenting many cases surrounded by the most unfavorable circumstances, and performed in the very worst possible manner. The statistics of other operations, of less danger and importance, are made up on the contrary, from the practice of men of good surgical reputation, surrounded by as favorable circumstances as experience of long standing, and innumerable repetitions can suggest. With regard to the statistics of ovariotomy, they differ very much, comparing those of the last ten years with the records of the twenty years preceding; and there is reason to hope, that twenty years hence, the progressive improvement of this operation, will furnish a still more favorable exhibit, as compared with the present.

Notwithstanding all this unfavorable state of things, ovariotomy will compare favorably with amputations, operations for hernia, ligations of large arteries, etc. I shall give the results of other operations before inquiring into those of ovariotomy: M. Malgaigue has shown that, out of 825 amputations of the extremities, of all kinds, including the fingers and toes, performed in the Parisian hospitals from the year 1836 to the year 1844—332 died; or about *four* in every *ten* proved fatal.

| | | | | |
|---|---|---|---|---|
| Of 201 | amputations of the | thigh, | 126 died, | or *six* in *ten*. |
| Of 192 | " " | leg, | 106 died, | or *five and a half* in *ten*. |
| Of 91 | " " | arm, | 41 died, | or *four and a half* in *ten*. |

In Glasgow, Dr. Lawrie has shown that of 276 amputations, 101 proved fatal, or nearly *four* in every *ten*.

| | | | | |
|---|---|---|---|---|
| In 128 | amputations of the | thigh, | 46 died, | or *three and a half* in *ten*. |
| In 62 | " " | leg, | 30 died, | or about *five* in *ten*. |
| In 53 | " " | arm, | 21 died, | or *four and a half* in *ten*. |

In the Edinburgh Infirmary during 1839, there were seventy-two amputations of the thigh, leg, shoulder-joint, arm and forearm; of these, thirty-five proved fatal,—nearly *five* in every *ten.**

Amputations at the knee-joint, show a mortality of thirty-seven per cent., as shown in a collection of cases by Dr. Thomas M. Markoe, in the New York *Journal of Medicine and Surgery*, for January, 1856, quoted by Dr. Gross, in his new work on Surgery, at p. 1119, second vol. In amputations of the thigh, performed by American and European surgeons, in 1055 cases, there were 464 deaths, or forty-three and a half per cent.; seventy-six cases in 126 amputations at the hip-joint, died, equal to about sixty per cent. In forty-seven cases performed for injury, thirty-five died, over *seventy* per cent.

Mr. Phillips has collected 171 cases in which the large arteries were tied; of these fifty-seven died, or about *three and a half deaths* to *ten* cases. Dr. Inman finds that, in 171 cases of ligature of the large arteries, sixty-six died, about *three and a half* to *ten*. Eighteen cases of ligature of the subclavian in forty died, nearly *five* to *ten*.

In his work on hernia, Sir Astley Cooper records thirty-six deaths in seventy-seven operations; or nearly *five* in *ten* cases. Dr. Inman has collected 545 cases of operations for hernia, of which 260 proved fatal, nearly *five* in *ten*.

Turning to operations in ovarian diseases. According to Mr. Southam, who has tabulated twenty cases of simple tapping, four died from the effects of the first operation; *one* in *five*. Three more died within one month of the first tapping, and fourteen, in all, died in less than nine months, and four lived during periods, varying from four to nine years.†

Dr Tyler Smith, in a paper read before the Obstetrical Society of London, says, that in 130 cases of simple tapping, as many as sixty-nine died within one year, and 114 were known to have eventually died of the disease. Of 130 cases, treated with iodine injections, sixty-six died or refilled, and

*Simpson 1*st Series*, *page* 245, *Obstetrical Works.*

†Simpson *Obs't Works*, *page* 24, 1*st Series.*

sixty-four are said to have been cured. The ovary still remaining, the nisus of the disease is left behind.*

Dr. Smith reports twelve cases in which *he* tried iodine injections after tapping; two were successful; one of these he lost sight of in a short time, and consequently, could not say how long she remained well.—*Idem.*

I cannot lay my hand upon the tables at present, but Dr. Robert Lee, a strenuous opposer of the operation of ovariotomy, has collected 200 cases. His table shows a mortality of one in every $2\frac{18}{42}$, or less than five in ten. Many of these were operations performed many years ago. The imperfections of diagnosis, and want of knowledge of the best modes, and appropriate sort of cases, should be considered in connection with these results.

Dr. Cormak, who is also adverse to the operation, found that in eighty-nine cases, in which ovariotomy had been performed or attempted, thirty-four died, or nearly four in ten. In sixty-five cases in which the operation had been perfected, twenty-five, or between three and four in every ten died.†

Dr. George H. Lyman reports the results in 299 cases, collected by him, in a prize essay read before the Massachusetts Medical Society, quoted by Dr. Gross, in his new work on Surgery; of these, 179 recovered, and 120 died, a mortality of four in ten, or a little over.

Dr. W. Tyler Smith reports 183 deaths in 212 cases, promiscuously collected, and four successful in his own practice. In the *British Medical Journal*, for December, 1860, a table is given of twenty-two cases, said to embrace all the operations performed in the London Hospital, for the last three years; of these thirteen died and nine recovered. The following is a copy of the table.‡

---

**Lancet for May*, 1861—*Am. Reprint.*

†SIMPSON, *Obst. Works*, *1st Series. Page* 246.

‡From *American Journal for April*, 1861.

| No. | Hospital. | Operator. | Date of Operation. | Result. |
|---|---|---|---|---|
| 1 | Samaritan, | Mr. S Wells, | February, 1858 | Recovered. |
| 2 | Metropolitan Free, | Mr. Hutchinson, | August, 1858 | Recovered. |
| 3 | " " | Mr. Hutchinson, | August, 1858 | Died. |
| 4 | " " | Mr. Hutchinson, | September, 1858 | Died. |
| 5 | Samaritan, | Mr. S. Wells, | September, 1858 | Recovered. |
| 6 | Metropolitan Free, | Mr. B. Childs, | November, 1858 | Died. |
| 7 | Samaritan, | Mr. S. Wells, | November, 1858 | Recovered. |
| 8 | University Coll., | Mr. Erichsen, | November, 1858 | Died. |
| 9 | Samaritan, | Mr. S. Wells | January, 1859 | Died. |
| 10 | Guy's, | Mr. C. Forster, | February, 1859 | Died. |
| 11 | Metropolitan Free, | Mr. B. Childs, | February, 1859 | Died. |
| 12 | Samaritan, | Mr. S. Wells, | May, 1859 | Recovered. |
| 13 | " | Mr. S. Wells, | June, 1859 | Died. |
| 14 | " | Mr. S. Wells, | June, 1859 | Recovered. |
| 15 | " | Mr. S. Wells, | July, 1859 | Recovered. |
| 16 | " | Mr. S. Wells | October, 1859 | Recovered. |
| 17 | Westminster, | Mr. Holt, | October, 1859 | Died. |
| 18 | Samaritan, | Mr. S. Wells, | December, 1859 | Died. |
| 19 | " | Mr. S. Wells, | January, 1860 | Recovered. |
| 20 | London, | Mr. Curling, | February, 1860 | Died. |
| 21 | Samaritan, | Mr. S. Wells, | February, 1860 | Died. |
| 22 | Middlesex: | Mr. Nunn, | October, 1860 | Died. |

Mr. Spencer Wells, who has diligently and profitably studied the subject of ovarian disease had, it will be seen, twelve of these cases under his care. The recoveries in his cases, were eight in the twelve, or a mortality of three and a half to ten. Mr. Hutchinson had the only other favorable case. This is remarkable,—as Messrs. Erichsen, Curling, Nunn, and Forster, were among the surgeons in attendance upon the others; and it will hardly be regarded as improper, to express the opinion, that Dr. Wells' success was not accidental. While the mortality in his cases is so low, comparatively, in the other operations, it mounts up to nine deaths in ten cases,—certainly no approximation, to the average as arrived at, even by enemies of the operation.

Dr. R. Lee estimates the mortality at forty to forty-five per cent., Dr. Safford Lee, at thirty-three per cent., Dr. Spencer Wells, the same, as also Drs. Churchill, Druitt, Brown, Simpson, Clary and others

From the foregoing statistical comparison, (much of which presents the case as unfavorably for ovariotomy, as can be justified by the worst summary of the cases on record,) I think it may be inferred, that the fatality attending amputations at the hip-joint, thigh, and shoulder-joint, operations for hernia, ligature of the subclavian, and some of the other large arteries, is greater than occurs in ovariotomy. Yet, what surgeon would think of refusing his patients, under all circumstances, the chances promised by these operations?

Comparisons of dangerous operations, however, should not decide us for, or against, the propriety of admitting one or the other, into the list of credit and legitimacy; so that if ovariotomy were proven to be the most dangerous of operations, the question of admissibility would not, thereby, be settled against it. The question properly at issue is: *Is ovariotomy, under any given set of circumstances, the best remedy for ovarian growths?*

If certain conditions present themselves, in which no other remedy can be used with hope of success, and the course of the case is to a speedily fatal termination, the life of the patient is worth very little to herself, friends, or the public. If the patient is young, say less than twenty-five, is the certainty of living a life of misery, one year or less, better than one chance in three, or even one in two, of being entirely relieved of her disease, and placed upon a healthy footing, with a reasonable prospect of a long and useful life? Nobody would recommend ovariotomy for a disease that, according to our best judgment, would not approach a fatal termination under ten or more years, or has not already destroyed the hope of the sufferer of ever again enjoying health. But with ovariotomy, as with every other terrible remedy, it should not be suggested, until the surgeon has become convinced that it is *the only remedy.* Then the responsibility of refusing that remedy is greater than the risks connected with a timely, judicious and skillful operation.

That ovarian cystic dropsy is not always fatal, two per cent. of exceptions will not prove. That its course is rather rapid and continuously progressive, is almost as equally true. The operation, when successful—not fatal, in its effects, is a complete remedy, removing the nisus of the evil. It is not attended with any danger to the patient, the health being as good as ever; all her functions are complete, unless when both ovaries are removed.

This is more than can be said of many important and dangerous operations. Amputations always mutilate and cripple their subjects, lessening the value of the lives they save; not so with ovariotomy. Many of the objections used against this operation, are equally applicable to every other, without exception, of the capital operations. That there are instances of mistakes in diagnosis is true. How many limbs, and lives together, have been sacrificed unnecessarily, on account of wrong diagnosis? Did no man of superior abilities and experience ever operate for aneurism or hernia, when neither of these existed? Because errors of diagnosis might more readily occur at one time, years ago, when our means of distinguishing between diseases of the abdomen and pelvis were very much inferior to what they are at present, should we be deterred from acting upon our convictions now, particularly in cases otherwise hopeless? Because we have means of less hazardous nature, that will enable us to give the patient some chance of relief in certain forms of ovarian disease, such as injections of iodine, tapping, and pressure, are we to abandon the rest of the cases, such as these remedies avowedly cannot reach, to their fate? I take it for granted, that no man would hope to cure a polycystic ovarian tumor by any other means than extirpation.

Inability to complete an operation, after being begun, on account of adhesions, the unexpected nature or complication of the tumor, is urged as an objection. When we recur to the facts, that we are justified in performing ovariotomy only in cases where it affords us the best chances of cure; and that without cure, our patient must die in a short time; and that proper tentative procedure, will enable us to decide the propriety or possibility of completing our operation, without adding very largely to the *immediate* risks of our patient, the objection will not be a very important one.

That all capital operations are too frequently and unnecessarily resorted to, that they are sometimes productive of avoidable mischief, no doubt are facts; but it is equally true that thousands of lives are annually saved by their performance, throughout the civilized world. The abuse of them should not be brought as an objection to their employment; nor should their want of success, from the unskillful practice of the ignorant, who may choose to undertake their performance, or from the evident imperfections of the various steps composing them, and the antecedent and after-treatment of the cases, discourage us. While there is life to be saved, as conservators of that greatest of human blessings, we should, at least, offer our patient even these extreme remedies.

Ovariotomy may be urged as one of the remedies capable of accomplishing, what, under certain circumstances, no other human agency can effect. No doubt, therefore, ought to be entertained of the propriety of recommending it, under such circumstances. Many of the most daring and deserving of our living surgical teachers take the opposite ground; but that ovariotomy is far more generally countenanced now than heretofore, is apparent to the intelligent observer; and at the end of the present century, there will be no more doubt of the propriety of the operation, than there now is of the admissibility of amputations of the thigh and shoulder. There is one circumstance, in connection with the propriety of substituting or temporarily using, other remedies for ovariotomy, in cases where this operation is proper, that has been, I think, too carelessly considered; and that is, that almost every other remedy we can make use of, with a view of producing any good palliative effect upon the tumor, renders the operation more hazardous, and sometimes impracticable. This is particularly true of tapping, either with or without injections of iodine, and pressure. These, and more particularly the last two, almost invariably produce peritoneal inflammation, effusion of fibrin, and consequent adhesions,—one of the most vexatious and dangerous complications with which we meet.

Hence, I think much of the fatality of ovarian diseases would be avoided, by a determination, before any interference whatever, of the propriety or impropriety of ovariotomy. Let each case, when first met with, be carefully considered with reference to the remedy applicable to it, and *make use of that remedy alone.* Doubtless, many cases would be presented, to which the palliative treatment is the only one applicable, as in incurable cases; in such cases it should be used *from*

*beginning to end. I do not believe it should ever precede radical remedies.*

We are too apt to think our responsibility begins with the spilling of blood in a doubtful operation; but much more attaches to a judicious decision, as to proper means, and an abstinence from mischievous but, apparently, unimportant meddling.

